# Experiences and challenges faced by community mental health workers when providing care to people with mental illness: a qualitative study

**DOI:** 10.1186/s12888-022-04252-z

**Published:** 2022-09-21

**Authors:** Chaoyang Li, Fen Yang, Bing Xiang Yang, Wencai Chen, Qinyu Wang, Haishan Huang, Qian Liu, Dan Luo, Xiao Qin Wang, Juan Ruan

**Affiliations:** 1 Hubei University of Chinese Medicine, School of Nursing, Wuhan, China; 2 Wuhan University, School of Nursing, Wuhan, China; 3 Wuhan Mental Health Center, Wuhan, China; 4 Department of Psychology, The Fourth Affiliated Hospital of Xinjiang Medical University, Urumqi, China; 5 Huazhong University of Science and Technology, Tongji Hospital Affiliated to Tongji Medical college, Wuhan, China

**Keywords:** Challenges, Community mental health services, Community mental health workers, Experiences, Mental illness, Qualitative study

## Abstract

**Background:**

Mental illness is a major burden of disease worldwide. Community Mental Health Services (CMHS) are key to achieving community-based recovery for people with mental illness. In China, even though the community management of patients with mental illness is improving, the barriers faced by Community Mental Health Workers (CMHWs) are unclear. This study explores the difficulties and challenges in CMHS from the perspective of CMHWs. The results of this study may provide a practical basis for the training of CMHWs.

**Methods:**

We carried out a qualitative study using an empirical phenomenological approach. Nine CMHWs were recruited from nine communities in Wuhan, Hubei Province, using purposive and snowball sampling. Face to face semi-structured in-depth interviews were conducted with them from December 27 to 28, 2019. Interview recordings were converted to text content by Nvivo 11.0 software and analyzed using Colaizzi’s phenomenological method.

**Results:**

Three main themes were identified in this study: 1) Lack of role orientation leads to role ambiguity, 2) Failure to establish a therapeutic trust relationship with patients, and 3) Lack of communication and collaboration with various departments and peers. Seven sub themes were also identified. In these themes, CMHWs emphasized the importance of role clarity, therapeutic trusting relationships, and effective communication and coordination mechanisms.

**Conclusion:**

Although China has made great efforts on the road to improving the quality of CMHS, several salient issues regarding CMHWs must be addressed to optimize the quality of services provided by CMHWs. Community mental health institutions should help CMHWs overcome these difficulties, by maximizing its value and promoting the development of CMHS.

## Introduction

Mental health has become a growing public health concern worldwide [1]. It has profoundly impacted millions of people’s lives, socially and economically [2–4]. Mental health is included in the United Nations 2030 Agenda for Sustainable Development and the World Health Assembly’s extension of the World Health Organization’s (WHO) Action Plan for Comprehensive Mental Health to 2030 [5]. According to the WHO assessment of disability adjusted life years (DALYs), mental illness is also a major disease burden in China, accounting for about 20.00% [6, 7]. Data from the China Mental Health Survey show the lifetime prevalence of any mental illness in the Chinese general population is 16.60% and the 12-month prevalence is 9.30% [8]. Since mental health resources in China are concentrated in large psychiatric hospitals in large eastern and central cities [9]. There are many people with mental illnesses who are not effectively addressed in terms of treatment and assistance and service management [10, 11]. This poses a huge challenge for mental health services in China [12]. In addition, more than 90.00% of people with mental illness in China live in the community receiving home and community-based management [13]. Therefore, it is crucial to build mental health services in the community.

Community Mental Health Services (CMHS) is a common development in mental health services worldwide [14]. It positively affects reducing to utilize other services, reducing stigma, improving help-seeking attitudes, and promoting mental health [15–17]. Many Central and Eastern European countries have been working to develop CMHS [18]. In the 1960s and 1970s, the “deinstitutionalization” movement and the “biopsychosocial” model of medicine emerged in Europe and the United States. As a result, Chinese CMHS has gradually evolved from pure disease prevention and treatment to a model that combines disease prevention and treatment with community rehabilitation [19]. Later, China established a three-tier network for the prevention and treatment of mental illness, with mental health hospitals, psychiatric departments of general hospitals, and mental health departments of community health service centers as the mainstay. A mental illness management system in China was also established with medical institutions as the backbone, community-based, and family-supported [19]. The community provides specialized and targeted mental health services for patients under the supervision and technical guidance of higher-level mental illness professional institutions [20]. China has also introduced many laws, rules, and work codes to improve the quality and effectiveness of CMHS, strengthen the mental health services, and promote mental health services in the community [21, 22].

The CMHWs are the main force behind CMHS in China. It is known that CMHS teams in Western countries have professional mental health nurses, psychiatrists, and counselors [23–25]. However, most CMHS in China are done by clinically related professionals, such as clinical medicine, public health, pharmacy, and nursing [26]. Most of them lack the psychological background and mental health professional training, and part-time employment in outpatient and management positions is common [26]. They need to be responsible for basic prevention and treatment, such as mental health knowledge spread, screening, medication guidance, condition observation, and referral for treatment. Second, they are also responsible for health management work such as information management, follow-up assessment, classification intervention, and health examination of patients with mental illness [19]. As one can well imagine, CMHWs may face many difficulties and challenges from different levels in their work in CMHS [19, 27]. These challenges include social stigma, negative feelings of families and patients, a significant imbalance between the number of CMHWs themselves and the workload of the position, and poor quality of services [28–31]. These worsen patients’ conditions and contribute to burnout and turnover among CMHWs [32–34]. Previous qualitative studies have mostly explored the potential value and practical experience of CMHWs in CMHS from the perspective of the patient or family [28, 29, 35–38]. It is important and necessary to find out the challenges of CMHS in China from the perspective of CMHWs.

This study aimed to explore the experiences of CMHWs from their perspective at different levels of their own, with their patients, and with their organizations. Insights from our results are envisioned to guide further improvements in service quality and professional training for CMHWs.

## Methods

### Design

We carried out a qualitative study through face-to-face semi-structured interviews. An empirical phenomenological approach was used to get detailed descriptions of CMHWs about their experiences [39]. The essence of the empirical phenomenological approach is to promote a direct grasp of things and to seek to understand things without any preconceived knowledge or bias. It focuses on commonality in describing the experience of the whole population. This study used the Consolidated Criteria for Reporting Qualitative Research (COREQ) as a guide for writing [40].

### Participants

Participants were recruited through purposive and snowball sampling. They are CMHWs from nine different communities in Wuhan, Hubei Province. Three participants declined to participate in this study because of work commitments. The sample size was determined by data saturation-that is, no new themes emerged from the participants’ experiences. The inclusion criteria for the study population were: 1) workers involved in CMHS, and 2) voluntary participation and informed consent. Exclusion criteria were: 1) participation in other studies during this study.

### Data collection

Data were collected through face-to-face personal semi-structured interviews with participants by two experienced researchers. A comprehensive review of the literature on CMHS was conducted prior to the start of the study. The research team initially developed an interview outline based on the purpose of the study, which was revised after pre-interviews and discussions. Formal interviews were conducted with 9 participants from December 27 to 28, 2019. With the consent of the participants, all interviews were audio-recorded for 20 ~ 30 minutes. To capture more comprehensive information, the researchers asked follow-up questions, clarifications, and repetitions based on the content of the interviews.

The core content of the interviews was as follows. 1) What do you think is the role and function of CMHWs in CMHS? 2) What difficulties have you encountered as a CMHW? Please give an example of how you solved it. 3) What was the greatest difficulty you encountered in your follow-up visits? 4) What do you think would be helpful in your practical work? What else would you like to add?

### Data analysis

The researchers transcribed, organized, and repeatedly read the interview recordings within 24 hours of the interviews using NVivo 11.0 software. They then used Colaizzi’s seven-step analysis method to analyze the collated textual material [41]. First, two researchers carefully read the interview transcripts, parsed out significant statements, and coded recurring, meaningful ideas. They then assembled the coded ideas, wrote detailed, omission-free descriptions, identified similar ideas and sublimated thematic concepts, and returned them to the interviewees for verification. Finally, all researchers on the research team discussed and revised the preliminary results to ensure accuracy of the results.

### Trustworthiness

Several strategies were used to ensure trustworthiness. Trustworthiness was obtained through in-depth interviews and peer debriefings. The two co-authors independently analyzed the transcripts by bracketing data with preconceived ideas and strictly following the modified Colaizzi’s method described above. The research team then compared and discussed the findings until a consensus was reached on themes, thematic groups, and categories. In terms of transferability, the researchers determined this by considering the research context and the participants’ quotations gathered through in-depth interviews. An audit trail was maintained to ensure that all analysis steps could be traced back to the initial interviews.

### Ethical considerations

This study was approved by the medical ethics committee of Wuhan University (2019YF2032). Participants were explained the purpose and voluntary nature of the study and signed an informed consent form prior to each interview. Confidentiality is ensured by using numbers instead of names (e.g., P1, P2, etc.) and deleting identifiable information from the transcript. All audio and text records are kept on a password protected computer. Throughout the study, we followed the standard guidelines for qualitative research reports.

## Results

Table 1 shows the details of the 9 CMHWs. 9 CMHWs were from 9 communities in Wuhan, Hubei Province. Seven of them were from communities in urban areas and two were from communities in urban-rural areas; seven were female, and two were male; six were nurses, and three were physicians. The average age was 34.22 years. The average number of people in their jurisdiction was 47,138, and the average number of people with mental illness under their management was 177. A thematic duplication occurred during the interview with the seventh individual, and the next two CMHWs were then interviewed to confirm the thematic duplication.

**Table 1:** Characteristics of the participants (*N* = 9)

Coding	Age (years)	Gender	Original profession	Position	Work experience (years)	Community source	Community population	Patients being managed
P1	23	Male	Nurse	CMHW	0.2	urban	10,242	30
P2	32	Female	Nurse	CMHW	1	urban	53,000	41
P3	43	Male	Physician	CMHW	1	Urban-rural	90,000	537
P4	47	Female	Nurse	CMHW	5.5	Urban-rural	30,000	248
P5	27	Female	Nurse	CMHW	3	urban	20,000	76
P6	32	Female	Nurse	CMHW	1	urban	32,000	30
P7	24	Female	Physician	CMHW	1	urban	60,000	151
P8	50	Female	Physician	CMHW	44	urban	105,000	393
P9	30	Female	Nurse	CMHW	0.2	urban	24,000	86

Three major themes were identified: 1) Lack of role orientation leads to role ambiguity, 2) Failure to establish a therapeutic relationship with patients, and 3) Lack of communication and collaboration with various departments and peers. Detailed results can be found in Table 2.

**Table 2:** Themes and subcategories

**1. Lack of role orientation leads to role ambiguity** 1) Lack of expertise in mental health 2) Unclear role responsibilities **2. Failure to establish a therapeutic relationship with patients** 1) Inadequate response capacity 2) Failure to meet the needs of patients 3) Stigma of mental illness has led to concerns about staff safety **3. Lack of communication and collaboration with various departments and peers** 1) No effective transfer mechanism has been established between peers 2) Lack of effective and stable communication and coordination mechanism with various departments	

### Lack of role orientation leads to role ambiguity

Role definition plays a vital role in the work of CMHWs. It emphasizes the individual’s role competencies and the importance of role responsibilities and role rights. The CMHWs play an irreplaceable role in CMHS. Their wide range of responsibilities and lack of expertise make CMHWs ambiguous about their roles.

#### Lack of expertise in mental health

Several CMHWs reported that although they had taken on CMHS, they had not trained as mental health or psychiatric professionals. Besides, none of them were health care professionals in psychiatry. Therefore, they lacked enough expertise in mental health.

*P9:“I don’t think any of us CMHWs seem to be very professional. “*

*P8:“ What we know about rehabilitation is just an abstract concept, and we don’t know exactly what we should do.”*

*P7:“ I learned less about mental health. The family members were very uncooperative, and they felt that they were not getting substantial help.”*

*P1:“ I am a nursing major and have no expertise in psychiatry.”*

Despite being in the same medical profession, the expertise in psychiatry that CMHWs learn in school seems to be very superficial. The importance of psychiatric expertise seems to be overlooked. Shallow expertise is not enough to support CMHWs in providing more specialized services to their patients.

*P5: “I am a nursing major and have not studied psychiatric nursing and symptoms in depth before.”*

*P7: “Previously in school, the piece of psychiatric expertise itself was less learned and not studied in depth.”*

Three CMHWs interviewed indicated that they were required to work in multiple positions in addition to not being psychiatric professionals. They were required to take on a much larger workload. A significant imbalance between workload and competency may lead to burnout and reduced motivation among CMHWs. This will not be conducive to improving the quality and efficiency of CMHS.

*P5: “All of our staff are part-time, and there are no specialists.”*

*P7: “I am both a family physician and I also manage mental health services. But I manage mental health services only part-time. I manage 151 people with mental illnesses, and more than 200 people with chronic illnesses.”*

*P8: “Although I am nominally a CMHW, I also must do other work such as Physical examination for the elderly and planned immunization. I’m not just representing myself, at least I know Qiaokou District CMHWs are like this.”*

Due to the lack of mental health expertise, three CMHWs often felt that the help they provided to their patients was limited. However, two CMHWs still desired to provide more help to those in need.

*P6:* “*I was only able to help with blood pressure and blood glucose, and I really felt inside that I was not helping enough. I wanted to give the patients more assistance.”*

*P7: “The help I can provide is more just some psychological counseling for him. I wish I could give them more useful help.”*

#### Unclear role responsibilities

The specific responsibilities of CMHWs are defined and refined in the nationally promulgated codes of practice for CMHS. However, the nine CMHWs had different opinions about the roles they were required to perform. One group felt that their primary role was to follow up and supervise. Another group felt that the CMHWs were primarily responsible for patient rehabilitation, psychological counseling, and health coaching. They did not seem to have a clear and comprehensive understanding of their scope of responsibilities and priorities.

*P1:“I think the main role and function is to follow up regularly and then to supervise the medication.”*

*P5: “We mainly implement the rehabilitation of community patients. If there are impulsive or unstable patients in the follow-up, they can contact the police or superior institutions in time. Then it can better contribute to the stability of the community.”*

*P6: “We acted as health educators, medication instructors, comforters and liaisons.”*

### Failure to establish a therapeutic trust relationship with patients

According to the description of CMHWs, there is a clear sense of distance between CMHWs and patients in the CMHS. The CMHWs did not establish a therapeutic trust relationship with patients, resulting in many family members and patients not cooperating with CMHWs.

#### Inadequate response capacity

In practice, it is clear that CMHWs lack the skills and ability to deal with complex situations. When patients are uncooperative, instead of further providing the most beneficial solution for the patient, CMHWs choose to let things slide. This negative response may deepen the distrust of patients and their families in the ability of CMHWs to solve problems, leading to more patient disengagement from community management.

*P1:“Some patients do not want to have their blood drawn, so I cannot force him to have his blood drawn. If he really does not want to, I can only respect his choice. Some patients don’t want us to follow up with them at home, so I can’t force them to go to their homes.”*

*P3:“During our follow-up visits, we found that some patients could not be contacted, and I did not know how to solve the problem. Some patients are reluctant to admit they have a disease because of privacy issues. We can’t do anything for this group of patients.”*

#### Failure to meet the needs of patients

In addition, to support in terms of aid policies, patients want more help in terms of financial resources, material resources, and physical health to facilitate physical recovery. However, CMHWs cannot fully meet the needs of patients due to their professional capacity, time, and resource constraints. The diverse unmet needs of patients and their families contribute to their skepticism about the role of CMHWs.

*P4: “When policies can actually be implemented, they’re a little more cooperative. They want practical help, such as financial, medication, rehabilitation guidance and supporting policies.”*

*P6: “Some families want us to help their children find jobs, but the reality is that we have no way to give help. In addition, some patients like to talk to us, but we have limited time and energy to meet their communication needs.”*

#### Stigma of mental illness has led to concerns about staff safety

People with mental illness who do not receive standardized community management and professional treatment are at higher risk of violence than those without mental illness [13, 42]. However, not all types of mental illness may be associated with an increased risk of violence [43]. Currently, there is a general lack of mental health knowledge among the public, and news media reports of negative events often associate mental illness with violence, instability, and danger, reinforcing the public’s stigmatization of mental illness [44]. It has caused CMHWs to have strong concerns about their safety.

*P7: “I’m actually quite worried about security, and I hope someone will keep us safe when we go to the door. There are still many cases of psychiatric patients hurting and killing people, especially those who are schizophrenic. “*

*P9: “It seems that most of CMHWs are women, and I think there is a very big safety risk when following up with patients in their home.”*

### Lack of communication and collaboration with various departments and peers

According to the description of CMHWs, there is a lack of effective communication and coordination mechanisms between CMHWs and various departments (e.g., disability associations, neighborhood councils, public security bureaus, etc.) and peers. This problem increases the workload and difficulty of CMHWs and can lead to delays or termination of patient services.

#### No effective transfer mechanism has been established between peers

Some CMHWs indicated that the most significant difficulty they met in their work is the high mobility of patients, many of whom often change residences. In response to this situation, they spent much time and energy on cross-community follow-up. There seems to be a lack of an adequate transfer mechanism between peers. It makes CMHWs feel overwhelmed.

*P3:“My biggest difficulty is that the patient population is highly mobile and often not in their place of household residence. Because patients with slightly stable conditions in rural areas have to go out to work to make ends meet, and I don’t know how to solve this problem of face-to-face visits.”*

*P5: “One community was demolished, and the patients moved away. I must look for them everywhere, even the neighborhood committee cannot contact them. The police station can only provide an address, which makes our work more difficult.”*

#### Lack of effective and stable communication and coordination mechanism with various departments

The CMHWs have failed to establish effective communication and collaboration mechanisms with various departments in their community mental health service work. They felt that the community sector seemed unclear about their responsibilities, roles, and importance in CMHS. This makes patient information not shared promptly and more fragmented. Several CMHWs even stated that they sometimes could not access complete patient information.

*P5: “Sometimes when you meet people who are difficult and unfamiliar people, they will not give you the list of patients. The person in charge of the street disability association split up the list of patients I was given. I had to piece together the correct information myself, which I found quite difficult. “*

*P9: “I think it is difficult to cooperate with various departments in the community, for example, the Disabled Persons’ Federation, the Community Council, the Public Security Bureau and so on. They often do not understand or cooperate with our work. When we ask them for help, they simply provide the patient’s contact information to contact them by ourselves. We sometimes feel very aggrieved.”*

## Discussion

As front-line workers in CMHS, CMHWs have become an important bridge for patients to return from the hospital to their families and communities. They are responsible for the increasing improvement of CMHS. The Chinese government has made great efforts to fill the gap in CMHS and has achieved excellent results [45]. These efforts include the implementation of various support policies, the construction of a three-tier prevention and treatment network, the construction of a community mental health system, and the training of mental health professionals [19]. However, The CMHS still face many difficulties and challenges.

Several key areas were identified in this study. Among them, CMHWs are often ambivalent and ambiguous about their role in CMHS [46, 47]. Communities in Western countries have teams of mental health services staffed by professional mental health nurses, psychiatrists, and psychologists [23–25]. However, community physicians, nurses, and part-time social workers are the main providers of CMHS in China. Most of them not only have several jobs, but also lack professional knowledge of mental health, systematic learning, and practice [26, 30]. Combined with the high population density and far higher number of people with mental illness in China, numerous communities lack professional psychiatrists and caregivers [20]. There is a serious imbalance between the number of CMHWs and the workload of the position [30]. This leads to a situation where some of the work and responsibilities of CMHWs may be beyond their capacity, leaving them feeling overwhelmed and powerless. This feeling of powerlessness is associated with burnout and negative job outcomes, which can drive more turnover [48]. These aspects eventually lead to early termination or delay of CMHS [49]. A study by Karkkola et al. found that role clarity through higher autonomy and higher competence can impact subjective dynamics at work [50]. Therefore, CMHWs can increase their motivation by proactively clarifying their roles and responsibilities as well as systematically learning expertise. The community should actively organize higher-level psychiatric professional institutions to provide more professional guidance and targeted training for CMHWs.

Another critical area identified was the importance of therapeutic trust relationships, particularly regarding demand supply, coping capacity, and stigmatization. The existence and maintenance of trust is the basis for the provision of CMHS [51]. Establishing a trust relationship can reduce patients’ unwillingness to participate in CMHS to a certain extent [28, 39]. The CMHWs mentioned that patients have high expectations of CMHWs and that they desire emotional interaction in addition to their financial and physical needs. They also want understanding, empathy, and more health knowledge through effective communication [24]. Second, they lack positive and flexible coping skills and coordination in the face of various complex situations such as refusal of follow-up, medical examination, or follow-up visits. Mental illness is a disorder of varying degrees of cognition, emotion, and behavior due to disorders of brain function [52]. During a psychotic episode, the patient’s lack of recognition and control over their behavior may lead to violent incidents [13]. However, people with mental illness are at much higher risk of becoming victims of violence than those who commit violence [53–55]. Health care interventions can be effective in preventing violent behavior in people with mental illness [54]. More than 90% of people with mental illness in China still do not receive any professional treatment [11]. This, combined with media coverage of negative events that often associate mental illness with violence and danger, adds to the public’s prejudice and discrimination against mental illness [44]. The stigmatization of mental illness has led to more substantial concerns about the safety of CMHWs.

These negative emotions and behaviors may lead to delays in treatment, worsening of illness, and decreased quality of life and social participation of patients [31, 56–58]. Building a therapeutic and trusting relationship with patients in this situation can be difficult. Some researchers have argued that the relationship with patients is a core principle of client engagement in treatment [59]. The CMHWs can build strong therapeutic trust with patients by developing better communication and listening skills, encouraging patients to express their needs and improving their coping skills [28]. In addition to training in professional knowledge and practical knowledge, there should be ideological education. These will help CMHWs reduce prejudice, discrimination, and stereotypes about people with mental illness. CMHWs need to have an open and inclusive attitude toward their patients.

Finally, in this study, CMHWs also emphasized the value of communication and cooperation with various departments and peers. China has made great efforts to improve the quality of CMHS and promote the mental health of the whole people. The responsibilities of various departments and personnel in the community are defined in various policy documents [19, 21, 22]. They should cooperate to promote mental health services’ development jointly. However, Participants in this study said they were often not understood and supported when communicating with various departments or peers, which hinders the process of their service plan. Research shows that the lack of communication and cooperation will make all departments have insufficient understanding of their responsibilities and importance in mental health services, and there is no clear common goal [60, 61]. The CMHWs often find it difficult to obtain complete patient information and need to spend their time piecing it together. This is also the difficulty faced by CMHS in Singapore [49]. The lack of effective communication and cooperation makes the patient’s information cannot be shared in time and become fragmented. It increases the workload and difficulty of CMHWs [46]. If the community wants to develop a solid mental health service team, it needs communication and cooperation within and between organizations to establish a sustained and effective cooperative relationship. In this way, even in the case of a lack of resources, the community can meet the supply of mental health services [62]. The CMHS is not only a matter of personal interests. All departments within the organization should clarify their responsibilities, understand each other, share responsibilities, and jointly promote social welfare and the rehabilitation of patients with mental disorders. Otherwise, the development of CMHS may be hindered [26, 63].

### Limitations of study

A limitation of this study is that it focused only on the experiences of CMHWs. Therefore, future studies may consider examining the perspectives of other stakeholders, such as a systematic exploration at the individual, family, social, and environmental levels, to gain a more comprehensive understanding of the difficulties and challenges faced by CMHS. This will help better utilize the community’s role in preventing, treating, and rehabilitating mental illness and thus more effectively contribute to achieving the “Health China 2030” strategic goal.

## Conclusion

Although China has made great efforts on the road to improving the quality of CMHS, several salient issues regarding CMHWs must be addressed in order to optimize the quality of services provided by CMHWs. The results of this study firstly highlight the need for CMHWs to improve their professional competence to clarify their role. Secondly, they need to develop their communication and problem-response skills to build good therapeutic and trusting relationships with patients. Finally, they need to communicate better with their organizations to build ongoing effective partnerships and gain more support.

## Data Availability

Due to the privacy of the participants involved in the study data, the datasets generated and/or analyzed in the study are not currently publicly available but are available from the corresponding authors of this study upon reasonable request.
